# Utilizing hiPSC-derived oligodendrocytes to study myelin pathophysiology in neuropsychiatric and neurodegenerative disorders.

**DOI:** 10.3389/fncel.2023.1322813

**Published:** 2024-01-11

**Authors:** Gina Shim, Alejandra I. Romero-Morales, Srinidhi R. Sripathy, Brady J. Maher

**Affiliations:** ^1^Lieber Institute for Brain Development, Johns Hopkins Medical Campus, Baltimore, MD, United States; ^2^The Solomon H. Snyder Department of Neuroscience, The Johns Hopkins University School of Medicine, Baltimore, MD, United States; ^3^Department of Psychiatry and Behavioral Sciences, The Johns Hopkins University School of Medicine, Baltimore, MD, United States

**Keywords:** oligodendrocytes, stem cells, myelination, neuropsychiatric disease, neurodegenerative disease, neural organoids

## Abstract

Oligodendrocytes play a crucial role in our central nervous system (CNS) by myelinating axons for faster action potential conduction, protecting axons from degeneration, structuring the position of ion channels, and providing nutrients to neurons. Oligodendrocyte dysfunction and/or dysmyelination can contribute to a range of neurodegenerative diseases and neuropsychiatric disorders such as Multiple Sclerosis (MS), Leukodystrophy (LD), Schizophrenia (SCZ), and Autism Spectrum Disorder (ASD). Common characteristics identified across these disorders were either an inability of oligodendrocytes to remyelinate after degeneration or defects in oligodendrocyte development and maturation. Unfortunately, the causal mechanisms of oligodendrocyte dysfunction are still uncertain, and therapeutic targets remain elusive. Many studies rely on the use of animal models to identify the molecular and cellular mechanisms behind these disorders, however, such studies face species-specific challenges and therefore lack translatability. The use of human induced pluripotent stem cells (hiPSCs) to model neurological diseases is becoming a powerful new tool, improving our understanding of pathophysiology and capacity to explore therapeutic targets. Here, we focus on the application of hiPSC-derived oligodendrocyte model systems to model disorders caused by oligodendrocyte dysregulation.

## Introduction

Oligodendrocytes are essential in the development of the brain, guiding the placement of the voltage-gated ion channels, providing metabolic support to neuron development, and importantly, myelinating axons for faster conduction. In some neurological disorders the oligodendrocytes’ myelin degenerates and then fails to fully remyelinate. Demyelinating disorders are complex, multifactorial conditions that lead to profound nervous system dysfunction. Recently, the paradigm has shifted to studying the disruption of glial cells, such as oligodendrocytes and astrocytes, as potential causes of neurological dysfunction in several disorders, such as Multiple sclerosis (MS), Leukodystrophy (LD), Schizophrenia (SCZ), and Autism spectrum disorder (ASD), where dysmyelination is observed concomitantly or as the predecessor of neuronal dysfunction (Mighdoll et al., 2015; Birey et al., 2017; Inamura et al., 2018; Phan et al., 2020; Fortune et al., 2022).

Our current understanding of demyelinating diseases and the development of therapeutic approaches are primarily based on animal models and postmortem studies of affected patients. Animal models are generated using a variety of methods including the introduction of genetic variants, chemical manipulation, viral infections, or immunological activation. These approaches simulate the patient’s genetics and recapitulate symptomatology (Procaccini et al., 2015; Inamura et al., 2018; Winship et al., 2019). These animal models have the advantage of faster neurodevelopment compared to humans, and genetic similarities between mice and humans gives rise to similar biological features and diseases (Mouse Genome Sequencing Consortium et al., 2002; Semple et al., 2013). However, there are caveats concerning the over-reliance on animal models in translational research. Fundamental differences between humans and mice, such as higher cognitive functions, genetic distinction, and a lack of heterogeneity in mouse models often leads to failures in translation of therapeutic interventions that were developed using murine models (Zhao and Bhattacharyya, 2018). Unfortunately, studying brain disorders is complicated by the limited access to living human brain tissue. Therefore, the postmortem brain is most often studied and has the benefit of encompassing risk genetics and disease heterogeneity. However, postmortem samples only allow examination of the final stages of disease, which limits access to study early and intermediate stages of disease progression, which hampers our ability to unravel etiology. Additional limitations of postmortem samples include limited access, variability in the patient’s pharmacological use and treatment, and uncertain correlation between the deficit and heterogeneity of the symptoms (Raabe et al., 2019; Daviaud et al., 2023).

The limitations of the aforementioned models and postmortem samples hinder our ability to closely study the cellular and molecular mechanisms during temporally relevant periods in the disease process which limits the development of effective therapeutic approaches. In addition, the close cellular interactions between neurons and glial cells are a critical component that is necessary for proper modeling of pathophysiology and disease mechanisms. In light of these considerations, the advent of human induced pluripotent stem cells (hiPSCs) is now providing a compelling new avenue to model demyelinating disorders in a human context (Orack et al., 2015). hiPSCs are a powerful resource that allow adult somatic cells to be reprogrammed into pluripotent stem cells which can be differentiated into many different cell types for modeling human tissue and disease. With the somatic cell containing the genome of the patient, hiPSC-derived cells express phenotypes and morphology similar to that of the actual patient tissue without artificial initiation of the disease. These advantages provide a new model system that may improve our ability to understand disease mechanisms and develop patient-specific therapeutic approaches.

This review highlights the advantages of hiPSC-derived models in studying the cellular and molecular pathways associated with demyelinating disorders, and future therapeutic implications. We focus on how disrupted interactions between oligodendrocytes (OLs) and neurons underlie several neuropathological disorders, including MS, LD, SCZ, and ASD. We highlight the strength of using hiPSC-derived oligodendrocytes in studying demyelinating disease and explain how these two-dimensional (2D) and three-dimensional (3D) models of the brain can help us delve into the etiology and progression of these diseases.

## Oligodendrocyte fate specification in the developing CNS

Oligodendrocytes are the last neural cell type to be produced in the developing brain. The majority of our understanding of the process of oligodendrogenesis and maturation has been attained through studying rodent and chicken models. Oligodendrocyte progenitor cells (OPCs) are generated from the neural progenitor cells (NPCs) in sequential waves in two regions of the developing CNS: the spinal cord and the forebrain (Fogarty et al., 2005; Kessaris et al., 2006; Tripathi et al., 2011).

In the spinal cord, there are multiple waves of OPCs. The first wave is dependent on the morphogen sonic hedgehog (SHH) which induces the expression of the oligodendrocyte transcription factors 1 and 2 (OLIG1 and OLIG2) (Pringle et al., 1996; Rowitch, 2004). The expression of these transcription factors (TFs) is necessary for the establishment of the ventral progenitor domain (pMN) which gives rise to the spinal motor neurons (Ravanelli and Appel, 2015). OPCs from the pMN appear around gestational week 6.5 in humans and embryonic time (E)12.5 in mice (Bergles and Richardson, 2015), and migrate in all directions of the spinal cord. A second wave of OPCs arises from the dorsal progenitor domain, and is independent of SHH, but rather is dependent on fibroblast growth factor (FGF) (Cai et al., 2005). These dorsal-derived OPCs are less migratory and remain primarily in the dorsal half of the spinal cord (Mitew et al., 2014) replacing the ventrally derived OPCs and representing ∼20% of the OPC population in the spinal cord. Lastly, a final wave of OPCs differentiation occurs postnatally, generating OLs that associate and myelinate axons in the ventral and dorsal funiculi of the developing white matter (Bergles and Richardson, 2015).

Similarly, in the developing forebrain, OPCs are generated in sequential waves in a ventral-to-dorsal pattern. In mammalian development, OPCs arise from NKX2.1^+^ progenitors in the ventricular zone (VZ) of the ventral medial ganglionic eminence (MGE) at 7.5 weeks post-conception in humans and E12.5 in mice (Kessaris et al., 2006; Buchet et al., 2011). These progenitors migrate to the entirety of the developing telencephalon, including the cortex. The subsequent differentiation wave is generated from GSH2^+^ precursors in the lateral ganglionic eminence (LGE) and MGE; and preferentially migrate to the cortex (Kessaris et al., 2006). The final wave of OPCs emerges from the EMX1^+^ dorsal forebrain progenitors in the ventricular-subventricular zone afterward in a SHH-dependent manner (Shimizu et al., 2005; Kessaris et al., 2006; Rowitch and Kriegstein, 2010; Tong et al., 2015; Winkler et al., 2018).

Lineage tracing of the first OPC wave in the forebrain showed the replacement of this population by progenitors derived from the second and third waves of oligodendrogenesis (Kessaris et al., 2006; Bergles and Richardson, 2015). The mechanism for this elimination is not known. Recent findings suggest that a subpopulation of the OPCs derived during the first wave survives into postnatal life and form preferential synaptic connections with interneurons from the same embryonic origin (Orduz et al., 2019).

The OPC population remains proliferative throughout postnatal life, representing ∼5% of the neural cell population (Pringle et al., 1992; Chang et al., 2000; Dawson et al., 2003). These cells are uniformly distributed in the CNS and are capable of migrating into different areas of the CNS in response to demyelination or injury (Dawson et al., 2003; Dimou et al., 2008; Rivers et al., 2008). Adult OPCs have different rates of proliferation depending on their niche. White matter OPCs have a higher rate of proliferation and maturation capacity than the gray matter counterparts which are quiescent and remain in an immature state (Dimou et al., 2008). OPCs have the capacity to terminally differentiate into pre-oligodendrocytes (pre-OLs), these cells either progress into myelinating OLs or undergo apoptosis (Barres et al., 1992; Trapp et al., 1997; Back et al., 2002; Gil and Gama, 2023).

## Interactions of neurons and oligodendrocytes during brain development

**FIGURE 1:**
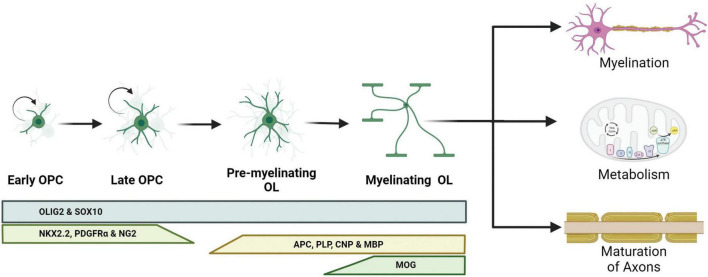
Schematic representation of oligodendrocyte differentiation and functions. Oligodendrocyte progenitor cells (OPCs) are capable of dividing and differentiating into premyelinating oligodendrocytes (pre-OLs). These pre-OLs either mature into myelinating oligodendrocytes (OLs) or undergo apoptosis. Different protein markers are expressed at these stages and can be used to identify the maturity of the OLs. The pan-OL markers oligodendrocyte transcription factor 2 (OLIG2) and sex determining region Y-box 10 (SOX10) are expressed throughout the OL life. OPCs are characterized for the expression of NK2 homeobox 2.2 (NKX2.2), platelet-derived growth factor receptor α (PDGFRα) and neural/glial antigen 2 (NG2). Committed pre-OLs express adenomatous polyposis coli (APC), proteolipid protein 1 (PLP), 2′,3′-cyclic nucleotide 3′ phosphodiesterase (CNP) and myelin binding protein (MBP); and myelinating OLs express myelin oligodendrocyte glycoprotein (MOG).

Oligodendrocytes are glial cells that specialize in myelinating the axons of neurons in the CNS, a process that increases the speed of propagation of the action potentials along the axon (Simons and Nave, 2015). Myelination occurs when the mature OLs extend their cell membrane, depositing a lipid and protein-rich coating around the axon in layers of insulation (Kuhn et al., 2019). Insulating sheaths increase membrane resistance along the axon and enable saltatory conduction, which increases action potential conduction velocity (Yamazaki, 2019). Conduction velocity is dependent on the amount of current escaping through the space between the axon and the innermost myelin wrap (Duncan et al., 2021). The variables of the interaction between oligodendrocytes and axons, such as the width of the space in between them, are regulated by neuronal activity during learning (de Faria et al., 2019). Myelination also allows for the efficient use of energy by reducing the capacitance of the axon and decreasing the energy required to restore the resting membrane potential after depolarization (Suminaite et al., 2019). As the neurons grow, oligodendrocytes affect the maturation of axons by localizing sodium and potassium channels into the nodes of Ranvier (NoR) (Buffington and Rasband, 2013). Therefore, proper neuronal development is dependent on interactions with oligodendrocytes (Figure 1).

### Sodium channel maturation

One of the key functions of myelin is forming the NoR, a distinct axonal domain that is critical for positioning voltage-gated sodium channels (Berret et al., 2016). Prior to myelination, sodium channels are regularly spaced on the axon, but as the myelin sheaths wrap the axon, oligodendrocytes secrete proteins that induce the clustering of sodium channels at the site of NoR (Kaplan et al., 1997). Studies have shown that conduction velocity increases along with increasing sodium channel clustering during development (Rasband et al., 1999). Experiments done to examine the effect of demyelination with or without oligodendrocyte death confirmed that myelin sheath regulates the clustering of sodium channels, but this regulation appears to be independent of myelin, as only the presence of oligodendrocytes is needed to maintain sodium channel clusters (Dupree et al., 2004). In humans and animal models of CNS demyelination, remyelination is capable of regenerating mature NoR (Prineas et al., 1993; Chang et al., 2002).

### Metabolic support

It has been proposed that independent of myelination, oligodendrocytes provide energy metabolites such as glucose or lactate to the axons of neurons (Lee et al., 2012). Oligodendrocytes are able to process glucose into pyruvate and export lactate to astrocytes and neurons which is called the astrocyte-oligodendrocyte-neuron lactate shuttle (Philips and Rothstein, 2017; Gil and Gama, 2023). The enhanced lactate in mice is shown to increase OPC differentiation (Ichihara et al., 2017). OL lineage-specific deletion of the lactate transporter monocarboxylate transporter 1 (MCT1), resulted in hypomyelination and axon damage with aging (Philips et al., 2021) and reduction in the expression of MCT1 is associated with amyotrophic lateral sclerosis (ALS) (Lee et al., 2012). Oligodendrocytes also provide energy in the form of glucose which is necessary for normal axonal function (Meyer et al., 2018). In addition to providing metabolic support to other cell types, oligodendrocytes themselves require a substantial amount of energy to generate membrane wraps for myelination. The inhibition of cytochrome c oxidase (COX), a crucial mitochondrial protein involved in the electron transport chain (ETC) (Diaz, 2010), results in delayed myelination, as well as a decrease in the population of OPCs and oligodendrocytes (Mahad et al., 2008).

## hiPSC derived oligodendrocyte brain models

**FIGURE 2:**
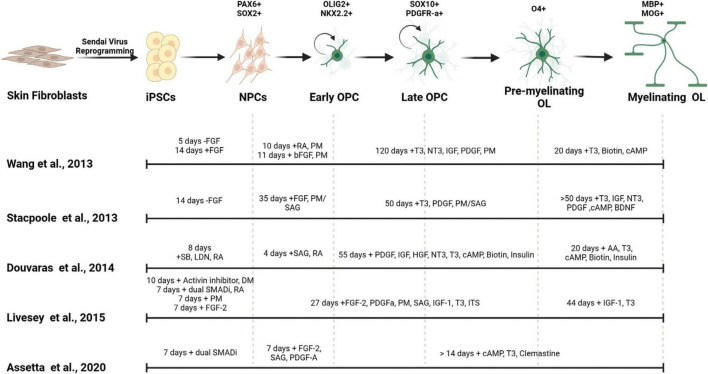
Generation of myelinating OLs from human derived iPSCs. Mature OLs can be generated from human derived tissues by reprogramming into induced pluripotent stem cells (iPSCs). Different small molecules and morphogens can be used to pattern the iPSCs into oligodendrocytic fate. The timing of the use of these components affect the cell identity, differentiation efficiency and maturity of the resulting OLs. FGF, fibroblast growth factor; SB, SB-431542; LDN, LDN193189; RA, all-*trans* retinoic acid; DM, dorsomorphin; PM, purmorphamine; SAG, smoothened agonist; T3, triiodothyronine; PDGF, platelet-derived growth factor; HGF, hepatocyte growth factor; IGF-1, insulin-like growth factor-1; NT3, neurotrophin 3; ITS, insulin-transferrin-selenium; cAMP, cyclic adenosine monophosphate; BDNF, brain-derived neurotrophic factor; AA, ascorbic acid.

There are two general methods, guided and unguided, to differentiate hiPSCs into neural cell types. Unguided differentiation allows the hiPSCs to follow their intrinsic developmental programs which leads to generation of a variety of neural cell types and brain regions. Guided differentiation pushes the hiPSCs to pattern into specific cell identities by controlling the environment with the addition of specific morphogens. Thus, directed differentiation typically produces a better representation of the brain region of interest, as it guides NPCs through specific developmental stages to become a particular cell type of interest such as excitatory neurons, interneurons, oligodendrocytes, and/or astrocytes. The majority of available established protocols for generating glial cells result in mixed cultures, composed of neurons, oligodendrocytes, and astrocytes and depending on the protocol the cell type of interest can be generated with a higher ratio to other neural cell types (Shaker et al., 2022). Below we discuss specific guided differentiation protocols that are developed to enrich cultures with oligodendrocytes (Table 1 and Figure 2).

**TABLE 1:** Summary of the different protocols that use pluripotent stem cells to generate oligodendrocytes.

	Wang et al., 2013	Stacpoole et al., 2013	Douvaras et al., 2014	Livesey et al., 2016	Assetta et al., 2020
Cell type	hESC and iPSCs	hESCs	WT and PPMS iPSCs	hESC, WT and ALS iPSCs	hESC and iPSCs
Protocol length	110–150 days	100 days + 5 weeks of OL maturation	75 days + 15 days of OL maturation	50 days + 30–45 days of OL maturation	2 weeks + 4–5 weeks maturation
Differentiation methodology	FGF2, PM, RA, bFGF, T3, NT3, IGF, PDGF-A	FGF2, PM, SAG, RA, PDGF-A, T3	Dual SMADi, RA, SAG, PDGF-A, NT3, T3, IGF, HGF, Biotin, cAMP, AA	Dual SMADi, RA, PM, SAG, PDGF-A, FGF, IGF, T3	Dual SMADi, PDGF-A, SAG, FGF, T3, cAMP, Clemastine
OL differentiation percentage	44–90% OLIG2+/NKX 2.2^+^ 4.0–15% O4^+^ 4.7–15.8% MBP^+^	61.6 ± 3.6% OLIG2^+^ 43.3 ± 5.4% O4^+^ 5.5 ± 0.9% MBP^+^ (from O4^+^)	44–70% O4^+^ 34 ± 4% MBP+ (from O4^+^)	∼13% PDGFRa+ ∼67.5% O4^+^ ∼89% MBP^+^ (from O4^+^)	>95% O4^+^ ∼25% OLIG2+
Myelination	MBP^+^ OL present. *In vivo* myelination in animal model	MBP^+^ OL present.	MBP^+^ OL present. *In vivo* myelination in animal model	MBP+ OL present.	MBP+ OL present in co-cultures with NG2 neurons
Transplant	Successful	N/A	Successful	N/A	N/A

### 2D models

#### Differentiation using small molecules

Recapitulating the process of generating human-derived oligodendrocytes on a dish was first accomplished by Nistor et al. (2005) using feeder-dependent human embryonic stem cells (hESCs). Non-directed neurospheres were generated, and an oligodendrocytic fate was determined by the addition of Triiodothyronine (T3 or thyroid hormone) and all-*trans*-retinoic acid (RA). The protocol generated OLs that successfully differentiated, integrated, and myelinated axons upon transplantation into the shiverer mouse model, which lacks the expression of myelin basic protein (MBP) and displays severe hypomyelination. Izrael et al. (2007) succeeded in generating highly branched mature OLs from hESCs *in vitro* by stimulating the levels of bone morphogenetic protein (BMP) in the progenitor cells through exposure to RA, which induced expression of NKX2.2, and then reducing BMP levels with the addition of Noggin, which induced SOX10 expression. These studies set the stage for multiple protocols that included the manipulation of different morphogens such as platelet-derived growth factor (PDGF-A) (Kang et al., 2007), SHH, and insulin growth factor 1 (IGF-1) (Gil et al., 2009; Hu et al., 2009; Sundberg et al., 2010; Piao et al., 2015). Further optimization of differentiation toward the OL lineage was achieved with the use of SMAD inhibitors (SMADi) to block the BMP and transforming growth factor beta (TGF-B) signaling pathways (Tomishima, 2008).

The first oligodendrocyte-specific protocol using hiPSCs was reported by Wang et al. (2013). This feeder-dependent protocol was tested in one hESC and three hiPSCs lines derived from different somatic sources. Neural fate specification was initiated via embryoid body (EB) formation without the addition of SMAD inhibitors. Terminal differentiation of the progenitors in this protocol generated both oligodendrocytes and astrocytes; and OPCs positive for SOX10, PDGFRa, and OLIG2 were observed after 110–150 days of culture. Transplantation of these OPCs into the shiverer mouse resulted in functional myelination by postmitotic OLs which increased the lifespan of the mutant mice (Wang et al., 2013). In that same year, Stacpoole et al. (2013) published a protocol showing differentiation of hESCs at 3% oxygen levels resulted in elevated yield of cell types from the OL lineage cells. They further demonstrated that the electrophysiological properties of these human OPCs were similar to those observed from rat OPCs, having the ability to generate action potentials and large voltage-gated sodium currents.

Further efficiency in generating the OL lineage was observed with the use of adherent cultures, dual SMAD inhibition, and retinoid supplementation starting from the beginning of hiPSC differentiation, yielding elevated numbers of OLIG2^+^ OPCs within 75 days (Douvaras et al., 2014; Douvaras and Fossati, 2015). The increased numbers of OPCs at early stages of differentiation from this protocol may be explained by the synergistic effect of activin/nodal receptor kinase inhibition and BMP4 inhibition via SMAD pathway inhibition, along with stimulation of the RA and SHH signaling. Subsequent PDGF-A withdrawal and ascorbic acid (AA) supplementation induced the differentiation of OPCs into OLs. Transplantation of OPCs generated from this protocol successfully recovered hypomyelination in the shiverer mouse brain. Moreover, Douvaras et al. (2014) generated hiPSCs from primary progressive MS (PPMS) patients and demonstrated patient-derived OPCs were capable of myelinating the shiverer mouse brain, suggesting that the disease mechanism is not directly associated with the intrinsic capacity of the OPCs to differentiate into OLs and myelinate axons.

The electrical membrane properties of OPCs and mature OLs were analyzed by Livesey et al. (2016). OPCs were generated by ventralization of the NPCs in the presence of RA and two SHH pathway activators: purmorphamine (PM) and smoothened agonist (SAG). Similar to the observations from Stacpoole et al. (2013), the maturation-specific physiological characteristics of OPCs and OLs was conserved between rodents and humans. Committed OLs exhibited a progressive decrease in voltage-gated sodium and potassium channels currents concomitant with an increase in inwardly rectifying potassium channel activity and a switch in AMPA receptor composition. In addition, this study used hiPSCs derived from a patient with ALS, and observed that patient-derived OPCs and OLs showed similar characteristics as cells derived from neurotypical individuals, suggesting that the mutations in *C9ORF72* does not appear to affect the maturation of the OL lineage (Livesey et al., 2016).

Most recently a variation on the Douvaras and Fossati (2015) protocol showed that supplementation of media with the promyelinating compound clemastine fumarate reduced the differentiation time of OPCs to OLs down to 28 days (Assetta et al., 2020; Mattingly and Chetty, 2023). Clemastine was previously identified as a promyelinating compound independent from its use as an antihistamine (Li et al., 2015; Liu et al., 2016; Green et al., 2017; Bohlen et al., 2023) and its ability to promote OPC differentiation appears to be through blockade of the M1 muscarinic acetylcholine receptor, which is a most common subtype of muscarinic acetylcholine receptor which regulates OPC proliferation and differentiations (Xiang et al., 2012; Chen et al., 2021; Palma et al., 2022).

#### Differentiation using transcription factors

Although guided OL differentiation using small molecules is a popular method used in the field, one downside of these protocols is the extensive time it takes to generate mature OLs. One way to enhance this differentiation timeline is to overexpress OL lineage-specific transcription factors (TFs) that are known to promote oligodendrogenesis. Overexpression of SOX10, OLIG2, and NKX6.2 in hiPSC-derived NPCs induced rapid differentiation of OLs (iOLs) (Ehrlich et al., 2017). This protocol generated cultures with 70% of the cells being O4^+^ OLs within 28 days and 3% of these O4^+^ cells being MBP^+^ within a week. Transplantation of these OLs successfully myelinated the shiverer mice.

In addition, overexpression of SOX10 alone is sufficient to generate O4^+^ and MBP^+^ OLs within 22 days from iPSC-derived NPCs (García-León et al., 2018). These iOLs demonstrated the ability to express MBP when engrafted into the shiverer mouse model, as well as when co-cultured with hiPSC-derived neurons. Moreover, this induction approach robustly generated iOLs from hiPSCs derived from MS and ALS patients. Induced differentiation of OPCs (iOPCs) directly from rodent and mouse fibroblasts has also been reported (Najm et al., 2013; Yang et al., 2013; Tanabe et al., 2022). This method utilizes expression of four transcription factors (OLIG2, SOX10, ASCL1, and NKX2.2) and generates iOPCs in 21 days that were capable of differentiating into mature OLs when transplanted into the shiverer mouse model. Moreover, this group showed iOPCs generated from Pelizaeus-Merzbacher disease (PMD) patients showed reduced survival compared to iOPCs generated from healthy donors.

### 3D models

Neural organoids are 3D cultures that model the complex features of the brain *in vitro* and allow the study of cell type- and stage-specific effects of neurodevelopmental and neurodegenerative disorders (Di Lullo and Kriegstein, 2017; Velasco et al., 2020). Unlike 2D models, 3D models recapitulate the cytoarchitecture and complex self-organization observed in the developing human brain. As these techniques are still relatively new, most of the protocols that generate these 3D structures are based on previous protocols established using mouse cells (Egawa et al., 2017; Flagelli et al., 2021) or by adapting the media composition and techniques from prior 2D protocols (Rodrigues et al., 2017; von der Bey et al., 2023). Guided organoid differentiation enriches certain cell types and can be used to study cell type-specific differentiation and the effects of mutations in a lineage-specific manner. These organoids can be generated by timed exposure to lineage-specific growth factors and hormones that encourage fate specification. OL enriched 3D models derived from stem cells are referred to as oligocortical spheroids (OCS), oligodendrocyte spheroids or myelinoids in the literature, based on their ability to generate larger oligodendrocytes to neuron ratio compared to other organoid protocols that are directed toward neuronal cell fates (Madhavan et al., 2018; Kim et al., 2019; Marton et al., 2019; James et al., 2021).

Oligodendrogenesis can be induced in dorsally patterned organoids by the supplementation of PDGF-A, IGF, and T3 to the media (Madhavan et al., 2018). This protocol is reported to have low interline and interspheroid variability, yielding between 18 and 21% myelin regulatory factor positive cells in the three different hiPSC lines by week 14. OPCs and myelinating OLs generated in this system can be used to screen compounds for their promyelinating potential and to investigate the effects of disease-causing mutations on the OL lineage.

Generation of both dorsal and ventral patterned organoids were shown to produce OLIG2^+^ NPCs that were capable of differentiating into OPCs and OLs (Kim et al., 2019). At week 5, the ventral forebrain organoids had a OLIG2^+^ yield of ∼50% whereas the dorsal counterparts had close to 10%. Moreover, when these dorsal and ventral organoids were fused, they observed an increased rate of OL maturation and active myelination of axons.

Ventral patterning and early induction of the OL fate generates spheroids that recapitulate multiple stages of OL development, migration, and myelination (Marton et al., 2019). Compared to 2D protocols, this approach generates MBP^+^ OLs as early as day 75, with the proportion of PDGFRa^+^ cells ranging between 25 and 46% between cell lines. Moreover, this protocol generates OPCs and OLs showing appropriate stage-specific properties, such as OPCs that display outward rectifying voltage-gated currents and spontaneous glutamatergic currents, while OLs show linear membrane properties and are capable of myelinating axons.

Most recently, James et al. (2021) demonstrated that early exposure to SHH agonists, RA, PDGF-A, and BDNF promoted the generation of mature OLs capable of generating compact myelin on axons with assembly of clear paranodal and nodal domains. This protocol is reported to resemble the development of the human spinal cord with mature OLs by day 59, yielding 18% MBP^+^CNP^+^/SOX10^+^ cells after 11–13 weeks in culture. In addition, they demonstrated their myeloid protocol was sensitive to *Nfasc155* deficiency, as patient-specific hiPSCs showed impaired paranodal axo-glial junction formation (James et al., 2021). Moreover, they were the first to show human OLs are capable of adaptive myelination by demonstrating that inhibition of synaptic vesicle release by tetanus toxin reduced the myelinating potential of the OLs.

### Advantages and disadvantages of hiPSC models

The use of hiPSCs to model oligodendrocyte development and disease is an exciting new direction that will increase our understanding of human OL development, neuronal-glial interactions and may improve translation by providing a human-specific approach to test promyelinating compounds and cell replacement therapies. Patient-derived cells allow for the study of species-specific mutations and human genetic heterogeneity, which are not possible in animal models. Thus, hiPSCs-derived OLs are a powerful emerging model system that has the potential to greatly expand our understanding of human OL development and function while also improving our disease modeling capabilities.

However, all cell models have their limitations. A major limitation of hiPSC-derived OL models is the lengthy time frame required to generate the OL lineage when using small molecules. These protocols closely mimic the lengthy time frame of OL development that is observed during *in vivo* human development. This extended time frame increases labor costs, slows experimental progress, reduces their potential to be scaled up for use in high-throughput screens, and can also increase the risk of epigenetic modification that can mask or confound observations.

Additionally, the long differentiation time frame in combination with the variability in the capacity of certain cell lines to generate the OL lineage is a concern. This issue is relevant in both 2D and 3D culture protocols, but may be exacerbated when using 3D protocols because of their lengthy differentiation times (Sivitilli et al., 2020). Another challenge that is specific to 3D models, is their lack of vascularization, which can limit the availability of oxygen and nutrients to the core of the organoid at later maturational stages (Giandomenico and Lancaster, 2017). Developing ways to overcome this issue is an active area of research on several fronts such as the addition of growth factors, *in vivo* transplantation into the mouse brain, and slicing the organoid to improve exposure of the core to the medium (Quadrato et al., 2017; Mansour et al., 2018; Giandomenico et al., 2019; Qian et al., 2020). Thus, the use of multiple clones from the same hiPSC genotype is recommended to rigorously validate observations, especially when modeling disease or performing drug screens.

## The use of iPSC-derived oligodendrocytes to study demyelination disease

Disruption of OL development and/or function is associated with a variety of disorders such as MS, LD, SCZ, and ASD (Mighdoll et al., 2015; Birey et al., 2017; Inamura et al., 2018; Phan et al., 2020; Fortune et al., 2022). A major focus in the field is to model these disorders using hiPSC-derived OLs to inform us about disease mechanisms that can be targeted for therapeutic intervention and for screening compounds for promyelinating potential. In addition, these models are being used to explore treatments based on restoring myelination through replacement of OLs. Below we focus on these neurological disorders and discuss progress in the use of hiPSC-derived OLs models to both inform us about disease mechanisms and for the development of therapeutic interventions.

### Multiple sclerosis

Multiple sclerosis is an auto-immune inflammatory neurodegenerative disease of the CNS causing brain atrophy and disabilities (Goverman et al., 1993). Early morphological studies of postmortem MS patients identified lesions devoid of myelination (Périer and Grégoire, 1965; Chari, 2007; Koutsoudaki et al., 2020). These demyelinated lesions cause inflammation which stimulates the cytotoxic T-cells to travel to the lesion and damage the neurons (Hafler et al., 2005). A key pathology of MS is that the damaged myelin cannot be quickly re-myelinated, exposing the axons to attack by the immune system (Fortune et al., 2022). Usually following demyelination events, resident OPCs regenerate myelination to restore the saltatory conduction and neurological function, but this process of remyelination declines with age and duration of illness (Chari, 2007; Koutsoudaki et al., 2020; Mozafari et al., 2020). Thus, it is critical to improve our understanding of the process of remyelination in MS so that effective therapeutic interventions can be developed.

Multiple sclerosis is a complex disorder with susceptibility being associated with a combination of genetic and environmental factors making it difficult to fully recapitulate in animal models (Gresle et al., 2020). A genome wide association study of MS identified 233 independent genetic variants associated with genetic risk for MS (International Multiple Sclerosis Genetics Consortium (IMSGC), et al., 2013), and thus the polygenic nature of MS severely limits the generation of animal models based on genetic risk. However, these challenges are beginning to be addressed with the use of MS patient-derived hiPSCs models which contain each individual patient’s set of common variants of risk (Fortune et al., 2022).

The first generation of MS patient-derived hiPSC lines werw reprogrammed through retroviral transduction of somatic cells from a female MS patient who was diagnosed with relapsing-remitting MS (RRMS) (Song et al., 2012). These first MS hiPSCs were shown to be capable of producing all three major neural lineages, and neurons derived from the patient line showed properties of functional neurons. Over the past decade, 52 MS patient hiPSC lines have been reported in the literature (Fortune et al., 2022). An early study using PPMS patient-derived hiPSCs showed that NPCs derived from these MS patients failed to rescue myelination in cuprizone-treated mouse models compared to NPCs-derived from neurotypical hiPSC lines (Nicaise et al., 2017). The failure of these PPMS-derived NPCs to rescue myelination appeared to be due to elevated cellular senescence (Nicaise et al., 2019). A similar increase in senescence was observed in PPMS hiPSC lines when differentiated into cerebral organoids, however this appears be specific to PPMS patient lines, as the senescence marker was not increased in hiPSC lines derived from relapsing remitting or secondary progressive MS (SPMS) patients (Daviaud et al., 2023). When MS patient-derived hiPSCs are directed to differentiate into the OL lineage, they perform similarly to hiPSCs derived from neurotypical individuals. For instance, the proliferation rate of OPCs, capacity to differentiate into OLs and ability to remyelinate the shiverer mouse model were all found to be comparable to control lines (Mozafari et al., 2020; Starost et al., 2020).

Additionally, a role of inflammation has been tested using hiPSCs from MS patients to determine if they are more sensitive to inflammatory signaling. The addition of the cytokines IFNγ and TNFα to control hiPSC-derived OL cultures was found to impair OL differentiation (Starost et al., 2020), and in a separate study, addition of IFNγ inhibited the differentiation of OPCs into OLs regardless of diagnosis (Morales Pantoja et al., 2020). However, some differences between the transcriptome, proteome and migration behavior of MS patient- and neurotypical-derived OPCs and OLs are reported (Lopez-Caraballo et al., 2020). Lopez-Caraballo et al. (2020) generated hiPSC lines from 4 patients diagnosed with secondary progressive MS (SPMS) and three neurotypical controls and differentiated these lines into the OL lineage by adapting the protocol of Wang et al. (2013). Although they observed no differences in the efficiency of OL lineage differentiation by diagnosis, they did observe many differentially expressed genes (DEGs) and secreted proteins between SPMS patient and control OPCs, with these DEGs and proteins converging on downregulation of pathways related to cell movement and migration. When cell migration was quantified, they observed that conditioned media from SPMS OPC cultures severely inhibited OPC migration, suggesting that abnormal migration of OPCs into areas of MS lesions may be a pathophysiological mechanism in MS.

### Leukodystrophy

Leukodystrophy is a group of related genetic disorders characterized by white matter degeneration that leads to a variety of symptoms including loss of motor function and cognitive decline (Parikh et al., 2015; Kevelam et al., 2016; Rutherford and Hamilton, 2019). It is estimated that there is over 50 different LDs caused by various monogenic mutations, with X-linked adrenoleukodystrophy (X-ALD), metachromatic LD (MLD), Krabbe disease (KD), Alexander disease (AD), and Aicardi-Goutieres syndrome (AGS) being the most common LDs (Stellitano et al., 2016). The white matter deficits observed in LDs are attributed to a failure of normal myelin formation or a progressive demyelination due to OL cell death. OL dysfunction is a critical pathophysiology in LDs, however many LD risk genes are expressed in other cell types beyond OLs, suggesting OL dysfunction can occur through either cell- or non-cell autonomous effects (Kevelam et al., 2016). For instance, AD is caused by mutations in the astrocyte marker gene *GFAP* (Leferink et al., 2018). Therefore, when developing models of LD using hiPSCs, consideration of cell types in relation to the specific genetic mutations being studied is required.

Although some animal models of LD can recapitulate aspects of the human pathophysiology observed in LD patients, many of these models fail to encompass important aspects of this human disease. Depending on the model, the timing of demyelination could be delayed or not occur at all, and generation of myelin phenotypes requires complete knockout of the gene of interest, which is not equivalent to the human genetics of LD (Rutherford and Hamilton, 2019). Fortunately, several studies using hiPSC-derived models of LD appear to be effectively overcoming these issues of face and construct validity present in animal models of LD.

Pelizaeus-Merzbacher disease (PMD) is a pediatric disease caused by mutations in the X-linked myelin gene proteolipid protein (*PLP1*). Numasawa-Kuroiwa et al. (2014) generated hiPSCs from two PMD patients carrying two different missense mutations in the *PLP1* gene. They differentiated these patient lines by adapting several prior protocols (Izrael et al., 2007; Kang et al., 2007; Hu et al., 2009) which generated mixed cultures containing neurons, astrocytes and oligodendrocytes. They observed no difference in the efficiency between a control line and the PMD lines to generate the OL lineage, however they did observe that PLP protein was devoid in the OL processes and was restricted to the endoplasmic reticulum. Because their cultures contained neurons, they performed transmission electron microscopy of ultrathin sections to quantify myelination. They observed PMD patient lines had fewer percentages of axons that were myelinated and axons that were myelinated had fewer numbers of myelin lamellae. Remarkably, they observed differences in the severity of these phenotypes between the two patient lines which correlated with the patient’s clinical severity, thus highlighting the potential effectiveness of hiPSCs to recapitulate clinical aspects of disease. Along these lines, Nevin et al. (2017), generated a panel of hiPSC lines derived from 12 PMD patients with varying mutations in the *PLP1* gene and a diversity of clinical phenotypes (Nevin et al., 2017). Using a 2D directed OL differentiation protocol (Douvaras et al., 2014), it was shown that PMD patient lines showed deficits in *PLP1* mRNA expression and splicing, OPC development, OL morphology and myelination capacity. Importantly, these phenotypes were somewhat variable depending on the patient’s particular *PLP1* mutation and their genetic background, again highlighting the usefulness of modeling clinical illness using patient specific hiPSC models.

Metachromatic leukodystrophy is a lysosomal storage disorder (LSD) caused by decreased levels of Arylsulfatase A (ARSA), an enzyme involved in desulfation of sulfatide, a specific sphingolipid (3-O-sulfogalactosylceramide) that is important for the development and function of OLs and Schwann cells (Marcus et al., 2006; Takahashi and Suzuki, 2012; Xiao et al., 2013; Grassi et al., 2016). MLD is characterized by progressive demyelination and dysfunction of the central and peripheral nervous system leading to rapid declines in motor and cognitive function (van Rappard et al., 2015). Mouse models of MLD indicate that sulfatide storage affects not only OL development but also neurons and astrocytes (Hess et al., 1996; Molander-Melin et al., 2004; Eckhardt et al., 2007; Ramakrishnan et al., 2007; van Zyl et al., 2010; Groeschel et al., 2012), however the extent of these phenotypes has not been confirmed in human models.

The first reported MLD patient-derived hiPSCs were generated from fibroblasts obtained from two MLD patients harboring different ARSA mutations (Meneghini et al., 2017). When these MLD patient lines were differentiated into the neural lineage altered sulfatide composition was observed along with expansion of the lysosomal compartment, increased oxidative stress and apoptosis (Frati et al., 2018). Similar to phenotypes observed in mouse models of MLD, patient lines showed delayed or reduced capacity to generate oligodendrocytes, astrocytes and neurons. Replacement of functional ARSA enzymes via lentiviral gene transfer normalized sulfatide levels and rescued these phenotypes, thus identifying a potential therapeutic approach for patients with MLD due to mutations in *ARSA*.

Krabbe disease, also known as globoid cell leukodystrophy, is a rare lysosomal storage disorder caused by deficiency of β-galactocerebrosidase (GALC) which is a catabolic enzyme of galactosphingolipids (Suzuki and Suzuki, 1970). KD is a childhood disorder, characterized by a total absence of myelin, severe gliosis, and the presence of multinucleated globoid cells in the white matter. More than 200 pathogenic variants are identified in the human *GALC* gene (Wenger, 1993) and KD patient derived hiPSCs were generated from five patients with distinct biallelic mutations in *GALC* gene (Mangiameli et al., 2021). All five KD hiPSC lines showed undetectable GALC activity and significant psychosine storage, which are hallmarks of KD. They then showed that lentiviral-mediated gene transfer of *GALC* could effectively rescue GALC enzymatic activity and abnormal psychosine storage. Depending on the disease-causing mutation, the neural progeny generated from patient lines displayed defects in OLs and neurons, unbalanced lipid composition, and early cellular senescence. However, only partial rescue of these phenotypes was observed in response to lentiviral replacement of *GALC* expression and caution was given about this rescue approach, as supraphysiological GALC levels produced unwanted effects on neural commitment and differentiation.

### Schizophrenia

Schizophrenia is a complex chronic mental disorder that has heterogeneous positive and negative symptoms (Birnbaum and Weinberger, 2017). Positive symptoms include auditory or visual hallucination, delusions, and disorganized thoughts and speech, and negative symptoms include anhedonia, alogia, affective flattening, apathy, and avolition. A complete understanding of the pathophysiology underlying SCZ is unresolved, with major hypotheses being focused on an imbalance of neurotransmitters, such as dopamine and glutamate (Howes and Kapur, 2009; Moghaddam and Javitt, 2012). However, neuroimaging studies and postmortem brain studies find evidence for defects in white matter and oligodendrocytes (Dietz et al., 2020). In addition, psychosis is observed in demyelinating diseases such as leukodystrophies and multiple sclerosis (Hyde et al., 1992; Walterfang et al., 2005; Maynard et al., 2021).

The polygenic nature of SCZ complicates our ability to model schizophrenia with animal models, as risk is associated with a genetic burden arising from an additive effect of common variants that are specific to the human genome (Trubetskoy et al., 2022). The recent identification of ultra-rare coding variants in 10 genes were shown to confer substantial risk for SCZ (Singh et al., 2022), and thus has created a potential opportunity for developing animal models based on these rare coding variants. One of the identified rare variant risk genes is *GRIN2A*, which encodes a subunit of the NMDA-type glutamate receptor. Interestingly, heterozygous and homozygous knockout of *Grin2a* in a mouse model lead to a variety of phenotypes in dopamine and glutamate signaling which aligned well with SCZ hypotheses. Intriguingly, differentially expressed genes were also found to be enriched in oligodendrocytes (Farsi et al., 2023). As data from mouse models of all of these SCZ rare variants become available it will be interesting to see how they compare and whether any convergent pathways or cell types emerge.

In order to model sporadic SCZ, patient-derived hiPSC models are an important strategy, as these cell lines contain all of the common variants which underlie each individual’s risk for developing SCZ. Several groups have begun to study SCZ using patient hiPSC lines to identify the effect of common variant risk on the development and function of neurons but so far only a few hiPSC study has differentiated oligodendrocytes from SCZ patient lines. Windrem et al. (2017) used iPSCs derived from SCZ patients to form OPCs and injected them into myelin-deficient shiverer mice. They observed that SCZ OPCs had more premature migration into the cortex compared to the controls which led to reduced white matter and hypomyelination (Wang et al., 2013; Windrem et al., 2017). McPhie et al. (2018) found that SCZ lines produce fewer OLs compared to control lines. They then correlated each individual’s myelin content measured from magnetic resonance imaging with the ability of their hiPSC line to produce OLs and observed a significant correlation, thus connecting clinical phenotypes to hiPSC-derived cellular measures (Brennand et al., 2011; Chen et al., 2017; McPhie et al., 2018). de Vrij et al. (2019) identified multiple rare missense mutations in the *CSPG4* gene (also known as NG2) that segregated with SCZ diagnosis. They generated hiPSC from *CSPG4* mutant carriers and non-carrier siblings and observe abnormal post-translational processing, aberrant subcellular localization of CSPG4/NG2, abnormal cellular morphology, reduced cellular viability, and impaired oligodendrogenesis. Moreover, using diffusion tensor imaging they observed mutation carriers showed reduced white matter integrity (de Vrij et al., 2019).

### Autism spectrum disorder

Autism spectrum disorder is a genetically heterogeneous disorder showing social communication deficits and repetitive behavior starting at an early age (Lord et al., 2018). The majority of ASD cases are complex and classified as sporadic, with genetic risk stemming from additive effects of common variants of small effect (Grove et al., 2019); however, approximately 10% of ASD cases are caused by rare and *de novo* variants of large effect (Coe et al., 2019; Kaplanis et al., 2020; Satterstrom et al., 2020; Fu et al., 2022). Although excitatory and inhibitory neurons have been a primary focus of ASD models, more and more evidence for defects in the OL lineage is beginning to appear. Phan et al. (2020) showed that convergent differentially expressed genes between three different ASD mouse models (*Tcf4*, *Pten*, *Mecp2*) were enriched for biological terms associated with oligodendrocytes and myelination and the eigengene (first principal component) of these 34 DEGs could be used to separate postmortem idiopathic ASD cases from control cases. With these same postmortem idiopathic ASD samples, they showed that cellular deconvolution predicted an increased proportion of OPCs and a reduction in OLs. Moreover, a single nuclei RNA sequencing study of postmortem brain from 33 ASD cases and 31 neurotypical controls found the OL lineage had the second most number of DEGs of all the cell types identified (Wamsley et al., 2023). Additionally, a spatial gene expression study of human dorsolateral prefrontal cortex observed previously identified differentially expressed genes from postmortem brain samples of ASD and neurotypical individuals were enriched in white matter (Maynard et al., 2021). Defects in the OL lineage as a component of ASD pathophysiology fits well with recent hiPSC studies using 3D organoids models of ASD, which show abnormal neurodevelopment in ASD is related to disruptions in progenitor cell fate specification (Phan et al., 2020; Paulsen et al., 2022; Jourdon et al., 2023; Li et al., 2023).

## Conclusion

In summary we highlight the potential of hiPSC models of the OL lineage to improve our understanding of myelin pathophysiology in neuropsychiatric disorders. The polygenic nature of many of these neuropsychiatric disorders severely limits the field’s ability to effectively model these disorders with animal models alone. Therefore the use of patient-derived hiPSCs is an important new approach which encompasses the patient’s entire genome and its associated genetic risk. We discuss four different neurological disorders, starting with two diseases, MS and LD, which have clear evidence for myelin pathology, followed by two disorders, SCZ and ASD, which have relatively unknown pathophysiology with less evidence supporting a role for myelin involvement. hiPSC-based studies of MS and LDs are clearly leading the way when it comes to modeling the OL lineage and expected phenotypes have been reported, thus giving us confidence in the use of hiPSCs-derived OLs to model myelin disease. As for hiPSC modeling of SCZ and ASD, we anticipate these models will soon inform us about how relevant myelin pathology is to the etiology and/or pathophysiology in these disorders.

## Author contributions

GS: Writing – original draft, Writing – review and editing. AR-M: Writing – original draft, Writing – review and editing. SS: Writing – original draft, Writing – review and editing. BM: Supervision, Writing – original draft, Writing – review and editing.
